# EEF1B2 regulates bone marrow-derived mesenchymal stem cells bone-fat balance via Wnt/β-catenin signaling

**DOI:** 10.1007/s00018-024-05297-x

**Published:** 2024-06-15

**Authors:** Shuhao Feng, Zihang Feng, Yiran Wei, Xiaoyong Zheng, Zhonghao Deng, Zheting Liao, Yangchen Jin, Ruge Chen, Liang Zhao

**Affiliations:** 1 Department of Orthopedics, Nanfang Hospital, Southern Medical University, No. 1838, North Guangzhou Avenue, Baiyun District, Guangzhou, Guangdong 510515 China; 2 Orthopaedic Department, The 4th medical center of Chinese PLA General Hospital, Beijing, 100089 China

**Keywords:** Mesenchymal stem cells, Osteogenesis, Adipogenesis, EEF1B2, Osteoporosis, β-catenin

## Abstract

The pathological advancement of osteoporosis is caused by the uneven development of bone marrow-derived mesenchymal stem cells (BMSCs) in terms of osteogenesis and adipogenesis. While the role of EEF1B2 in intellectual disability and tumorigenesis is well established, its function in the bone-fat switch of BMSCs is still largely unexplored. During the process of osteogenic differentiation, we observed an increase in the expression of EEF1B2, while a decrease in its expression was noted during adipogenesis. Suppression of EEF1B2 hindered the process of osteogenic differentiation and mineralization while promoting adipogenic differentiation. On the contrary, overexpression of EEF1B2 enhanced osteogenesis and strongly inhibited adipogenesis. Furthermore, the excessive expression of EEF1B2 in the tibias has the potential to mitigate bone loss and decrease marrow adiposity in mice with osteoporosis. In terms of mechanism, the suppression of β-catenin activity occurred when EEF1B2 function was suppressed during osteogenesis. Our collective findings indicate that EEF1B2 functions as a regulator, influencing the differentiation of BMSCs and maintaining a balance between bone and fat. Our finding highlights its potential as a therapeutic target for diseases related to bone metabolism.

## Introduction

Osteoporosis is a condition that affects the bones as people age, causing them to become weaker and more prone to fractures, even from minor injuries [1, 2]. The pathological changes of osteoporosis mainly include decreased mineralization and accumulation of marrow adiposity [3–5]. Bone marrow-derived mesenchymal stem cells (BMSCs) are progenitors that have the ability to self-renew and differentiate into osteoblasts, chondrocytes, and adipocytes [6–9]. The uneven differentiation of BMSCs into either osteoblasts or adipocytes plays a role in the development of osteoporosis [10, 11].

Protein synthesis requires the participation of eukaryotic elongation factor 1 (eEF1) [12]. The eEF1 family comprises eEF1A and eEF1B complex [12]. *EEF1B2* encodes the eEF1B proteins, which consist of the α, β, and γ subunits [13, 14]. Extensive research has been conducted on the role of EEF1B2 as a guanine nucleotide exchange factor (GEF) for eEF1A [15–17]. In addition to its role in translation elongation, previous research has demonstrated that EEF1B2 is expressed in various tissues and cell lines and at different developmental stages [18]. Moreover, EEF1B2 has been implicated in intellectual disability and tumorigenesis. EEF1B2 was found to be overexpressed in lung cancer in humans [19]. Conversely, intellectual disability is caused by the loss of function in the *EEF1B2* gene [20, 21]. Nevertheless, the expression of EEF1B2 in BMSCs and its function and underlying molecular mechanisms in the modulation of BMSCs differentiation are still poorly understood.

BMSCs exhibit a high degree of plasticity, allowing them to differentiate into various cell lineages. In vitro, the differentiation of BMSCs can be manipulated by adjusting the composition of the culture medium. For instance, exposure to an osteogenic induction medium containing factors β-glycerophosphate and ascorbic acid prompts BMSCs to differentiate into osteoblasts. Conversely, treatment with adipogenic components, including insulin, rosiglitazone, and IBMX, induces BMSCs to differentiate into adipocytes [22–24]. The differentiation of BMSCs is regulated by a complex network influenced by various factors, such as changes in the microenvironment, chemical factors, and non-coding RNAs like miRNA-128 and ANCR, which act through different molecular signaling pathways [25, 26]. The Wnt/β-catenin pathway has been identified as a key regulator of BMSCs differentiation [27, 28], with factors like Foxf1, Piezo1, Stat3 and CDC20 facilitating osteogenesis and adipogenesis of BMSCs by modulating β-catenin activity [29–32]. So far, the investigation of the regulator of β-catenin during BMSCs differentiation is still required.

This study shows that during osteogenic differentiation, the expression of EEF1B2 is increased in BMSCs, whereas it is decreased during adipogenic differentiation. In vitro, the suppression of EEF1B2 in BMSCs resulted in decreased osteogenic function and enhanced adipogenic specialization. Consistently overexpressing EEF1B2 in the BMSC cell line resulted in enhanced osteogenesis and suppressed adipogenesis. In vivo, the excessive expression of EEF1B2 can mitigate bone loss and decrease the amount of fat in the bone marrow of mice with osteoporosis. Mechanically, EEF1B2 controlled the transition from bone formation to fat formation in BMSCs by influencing the function of the Wnt/β-catenin signaling pathway. Our data identified EEF1B2 as a regulator of BMSCs in maintaining bone-fat equilibrium.

## Materials and methods

### Mice

The C57BL/6 mice were purchased from the Laboratory Animal Center of Southern Medical University. All animal experiments were approved by the Animal Care and Use Committee of Nanfang Hospital, Southern Medical University (IACUC-LAC-20230620-003) and were following the guidelines of the National Institute of Health. All animals were maintained in the animal facility of the Nanfang Hospital and housed under standard conditions of constant temperature and humidity on a 12/12 h light/dark cycle.

### Cell isolation, culture and differentiation

BMSCs were isolated from 6 weeks old C57BL/6 mice femurs and tibias bone marrow and cultured in α-minimum essential medium (α-MEM) containing 10% fetal bovine serum (FBS) and 1% penicillin and streptomycin (P/S, all from Gibco, Grand Island, NY, USA) in a 37 °C incubator with a 5% CO2 atmosphere. The adherent cells were digested and cultured until 80% confluence. Cells between passages 3 and 5 were utilized for the experiments, and all in-vitro experiments were repeated in triplicate.

The C3H10T1/2 cell line (#CL-0325) was purchased from Procell Life Science & Technology Co., Ltd. (Wuhan, China). The C3H10T1/2 cells were cultured in α-MEM containing 10% FBS, 2mM L-glutamine and 1% P/S (Gibco, Grand Island, NY, USA).

For osteogenic induction, BMSCs were cultured in osteogenic medium containing 50 µg/ml ascorbic acid and 10 mM β-glycerophosphate [24]. C3H10T1/2 cells were cultured in osteogenic medium containing 50 µg/ml ascorbic acid, 10 mM β-glycerophosphate, and 100 nM dexamethasone (all from Sigma-Aldrich, St. Louis, MO, USA). The culture medium change was performed every 3 days [33, 34].

For adipogenic induction, adipogenic medium consists of adipogenic medium A (AM-A) and adipogenic medium B (AM-B). AM-A consist of αMEM, 20%FBS, 1%P/S, 1µmol/mL dexamethasone, 10 µg/mL insulin, 1µM rosiglitazone and 0.5mM 3-Isobutyl-1-methylxanthine (IBMX). AM-B consist of αMEM, 20%FBS, 1%P/S, 1µM rosiglitazone and 10 µg/ml insulin. BMSCs and C3H10T1/2 cells were cultured in AM-A for 2 days, followed by AM-B for 4 days with culture medium changed every 2 days, to induce differentiation into adipocytes [24].

### siRNA-mediated knockdown and cell transfection

Eef1b2-specific siRNAs and negative control siRNA (NC) (RiboBio, Guangzhou, China) were used to cell transfection. Transfection of siRNA oligonucleotides was performed using Lipofectamine RNAimax (Invitrogen, Carlsbad, CA, USA) according to the manufacturer’s instructions. Eef1b2 expression was determined by quantitative reverse transcription PCR (RT-PCR). Transfected cells were passaged and used for downstream analyses.

### Alkaline phosphatase (ALP) and Alizarin Red S (ARS) staining

For ALP staining, Differentiated BMSCs were fixed with 4% paraformaldehyde (Solarbio, China) for 15 min. The cells were washed three times with PBS and stained with 1-Step nitro blue tetrazolium (NBT)/5-bromo-4-chloro-3-indolyl phosphate (BCIP) (Thermo Fisher, MA, USA) for 30 min and washed by PBS. ALP-positive cells were visualized by light microscopy or scanning.

For ARS staining, Differentiated BMSCs were fixed with 4% paraformaldehyde for 15 min. The cells were washed three times with distilled water and stained with ARS staining solution for 15 min. The staining solution was removed, and the cells were washed three times in distilled water. The mineralized part was visualized by scanning.

### Oil Red O staining

To detect the lipid droplet formation of BMSCs after adipogenic differentiation, cells were fixed in 4% paraformaldehyde for 15 min at room temperature, washed with PBS, and stained with an Oil red O staining kit (Solarbio, Beijing, China) for 20 min.

### Lentiviral construction and cells transfection

EEF1B2 overexpression lentiviruses were generated by Tsingke Biotech Co., Ltd (Beijing, China). The lentiviruses were then transfected into C3H10T1/2 (MOI:20) and screened with puromycin.

### Small molecule treatment

WAY-262,611 (Selleck, TX, USA) and ICG-001 (Selleck, TX, USA) were dissolved in dimethyl sulfoxide (DMSO) and administrated at a concentration of 3µM. Control groups received equivalent volumes of DMSO.

### RNA isolation and quantification of mRNA expression

TRIzol™ reagent (Thermo Fisher, MA, USA) was used to extract the total RNA from the cells. PrimeScript RT Reagent Kit (Takara, Otsu, Japan) was used to synthesize complementary DNA (cDNA). A qRT-PCR was performed by using an SYBR Green PCR Kit (Takara, Otsu, Japan) as directed by the manufacturer. Gene expression levels were analyzed relative to β-actin or GAPDH. The primer sequences are shown in Table S1.

### Western blot

Cells were lysed using radioimmunoprecipitation assay (RIPA) buffer (Solarbio, Beijing, China) with protease inhibitor mixture (Roche, Swiss). Total cell lysates were analyzed using Western blotting. Western blotting analyses were conducted using standard procedures. The details of the antibodies used are provided in Table S2.

### OVX animal model and AAV injection

Bilateral surgical ovariectomy (OVX) was performed to create a mouse model of osteoporosis in postmenopausal condition [35]. Briefly, 8 weeks old female mice were anesthetized. Then, the mice were subjected to Sham surgery or bilateral surgical ovariectomy by the dorsal approach. In the Sham operation group, the anesthesia, fixation, and selected incision were the same as those in the model group.

For AAV injection, 4 weeks after surgery, OVX mice were anesthetized. AAV-eGFP and AAV-EEF1B2 (Tsingke, Beijing, China) were injected into OVX mice tibias. In the Sham operation group, mice tibias were injected with PBS. µCT scanning analyses µCT scanning of mice tibias was conducted using a SkyScan1276 according to standard procedures and data were analyzed using software from the manufacturer.

### Hematoxylin and eosin (H&E) staining and immunofluorescent (IF) analysis

Tibias were fixed in 4% paraformaldehyde at 4 °C shaker overnight. Then they were decalcified by using EDTA solution at 4 °C shaker for 14 days. Those tissues were processed for either cryostat or paraffin sections. For H&E staining, paraffin sections were used with standard protocol. For IF staining, cryosections were rehydrated, washed with PBST (PBS + 0.05% Triton), and were blocked in 3% BSA (Bovine Serum Albumin) in PBST. IF staining were performed using standard methods. The details of the antibodies used are provided in Table S2. Sections were mounted in DAPI mounting medium (Vector laboratories). The staining was photographed with Zeiss Axio Imager D2 (Zeiss, German).

### RNA-seq and bioinformatics analysis

Total RNA of negative control siRNA (NC) or Eef1b2-targeted siRNA (siEef1b2) treated- BMSCs were extracted by SteadyPure Quick RNA Extraction Kit (Accurate Biology) following the manufacturer’s instructions 7 days after osteogenesis induction. Four biological replicates of each group were tested. Total RNAs were delivered to a facility core for quality proof, cDNA library construction, and RNA sequencing. High-throughput sequencing was performed using the Illumina Novaseq 6000 (USA). The RNA-seq reads were aligned to the mouse genome (GRCm39) using HISAT2 [36]. StringTie was subsequently used to count reads in features [37]. Genes of less than ten counts among all groups were filtered out using DESeq2 in R prior to the downstream analyses. Regularized logarithm (rlog) was applied to transform the read counts for Principal component analysis (PCA) and plotting [38]. Genes with Benjamini-Hochberg’s false discovery rate (FDR) < 0.05 and fold change > 2 were defined as significantly differentially expressed genes (DEGs) between conditions. The volcano plot was generated by the ggplot2 package in R [39]. Heatmaps were generated by the pheatmap package in R. Gene ontology (GO) analysis was performed using the R package clusterProfiler [40, 41], input with the down regulated genes in the siEef1b2 group. Enriched pathways were ranked based on the adjusted p-value calculated by the software. Gene Set Enrichment Analysis (GSEA) was performed using GSEA software (version 4.3.2) following the manufacturer’s instructions, input with normalized count matrix generated by the DESeq2 package in R [42].

### Statistical analysis

Data were shown as the mean ± standard deviation (SD). GraphPad Prism was used to conduct the analysis. To evaluate statistical significance, a two-tailed Student t-test was used to compare two groups, while one-way ANOVA analysis was employed for multiple comparisons. A statistically significant difference was indicated for all experiments when *P* < 0.05.

## Result

### EEF1B2 is increasingly expressed in BMSCs over osteogenic differentiation while decreasingly over adipogenic differentiation

**Fig. 1:**
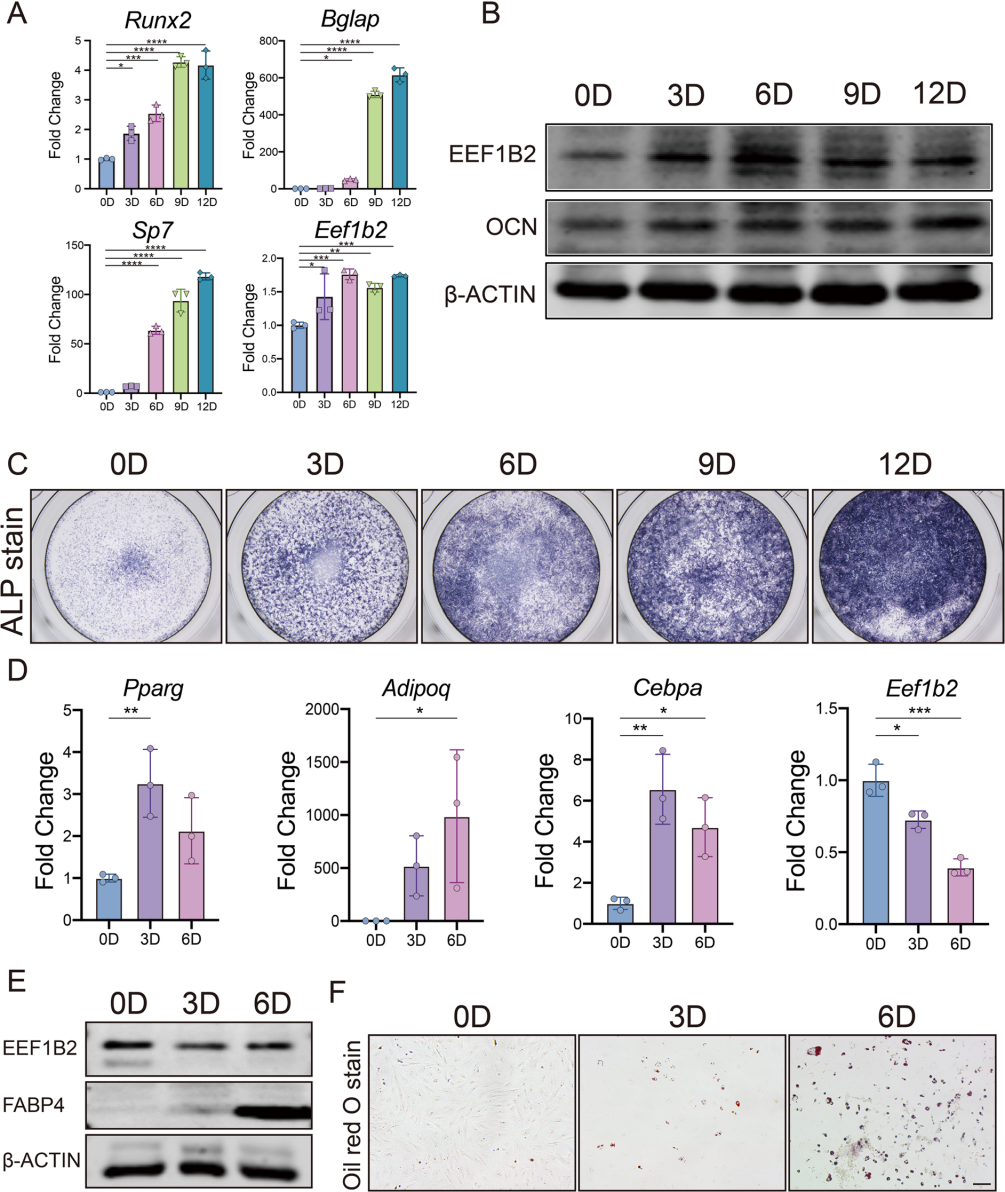
EEF1B2 expression is increased in osteogenic differentiation and decreased during adipogenic differentiation. **(A)** Relative expression of *Eef1b2* and osteogenic marker genes *Runx2*, *Bglap* and *Sp7* in mouse BMSCs during osteogenesis were assessed by qRT-PCR. **(B)** Western blot analysis of EEF1B2 and OCN protein levels during osteogenic differentiation of mouse BMSCs. **(C)** Representative image of ALP staining of mouse BMSCs on the indicated days of osteogenic differentiation. **(D)** Relative expression of *Eef1b2* and adipogenic marker genes *Pparg*, *Adipoq* and *Cebpa* in mouse BMSCs during adipogenesis were assessed by qRT-PCR. **(E)** Western blot analysis of EEF1B2 and FABP4 protein levels during adipogenesis of mouse BMSCs. **(F)** Representative image of Oil Red O staining of mouse BMSCs on the indicated days of adipogenesis. Scale bar, 100 μm. Data presented as mean ± SD. *P < 0.05, **P < 0.01, ***P < 0.001, ****P < 0.0001

To identify the role of EEF1B2 in BMSCs differentiation, we first investigated the EEF1B2 expression in mouse BMSCs. The expression of EEF1B2 in BMSCs is indicated by the results of qRT-PCR and western blot. The mRNA levels of *Eef1b2* and osteoblast markers *Runx2*, *Sp7*, *Bglap* (Fig. 1A) are increased in BMSCs during osteogenic differentiation, along with higher protein levels of EEF1B2, Osteocalcin (OCN)(Fig. 1B) and ALP (Fig. 1C). It was verified that the EEF1B2 expression is elevated throughout the process of osteogenic differentiation (Fig. 1A, B). Afterwards, we investigated the expression of EEF1B2 in BMSCs throughout the process of adipogenic differentiation. The expression of adipocyte markers in both mRNA and protein levels was enhanced when mice BMSCs were cultured in adipogenic medium (Fig. 1D, E). Oil O Red staining showed increased lipid droplet formation during the adipogenic differentiation process (Fig. 1F). Concurrently, the expression of EEF1B2 is reduced during the adipogenic differentiation of BMSCs (Fig. 1D, E). These results indicate that EEF1B2 might play an opposite role in osteogenic and adipogenic differentiation of BMSCs. EEF1B2 is potentially involved in the regulation of osteogenic-adipogenic differentiation balance of BMSCs.

### Suppression of EEF1B2 in BMSCs hampers the process of osteogenic differentiation and mineralization

**Fig. 2:**
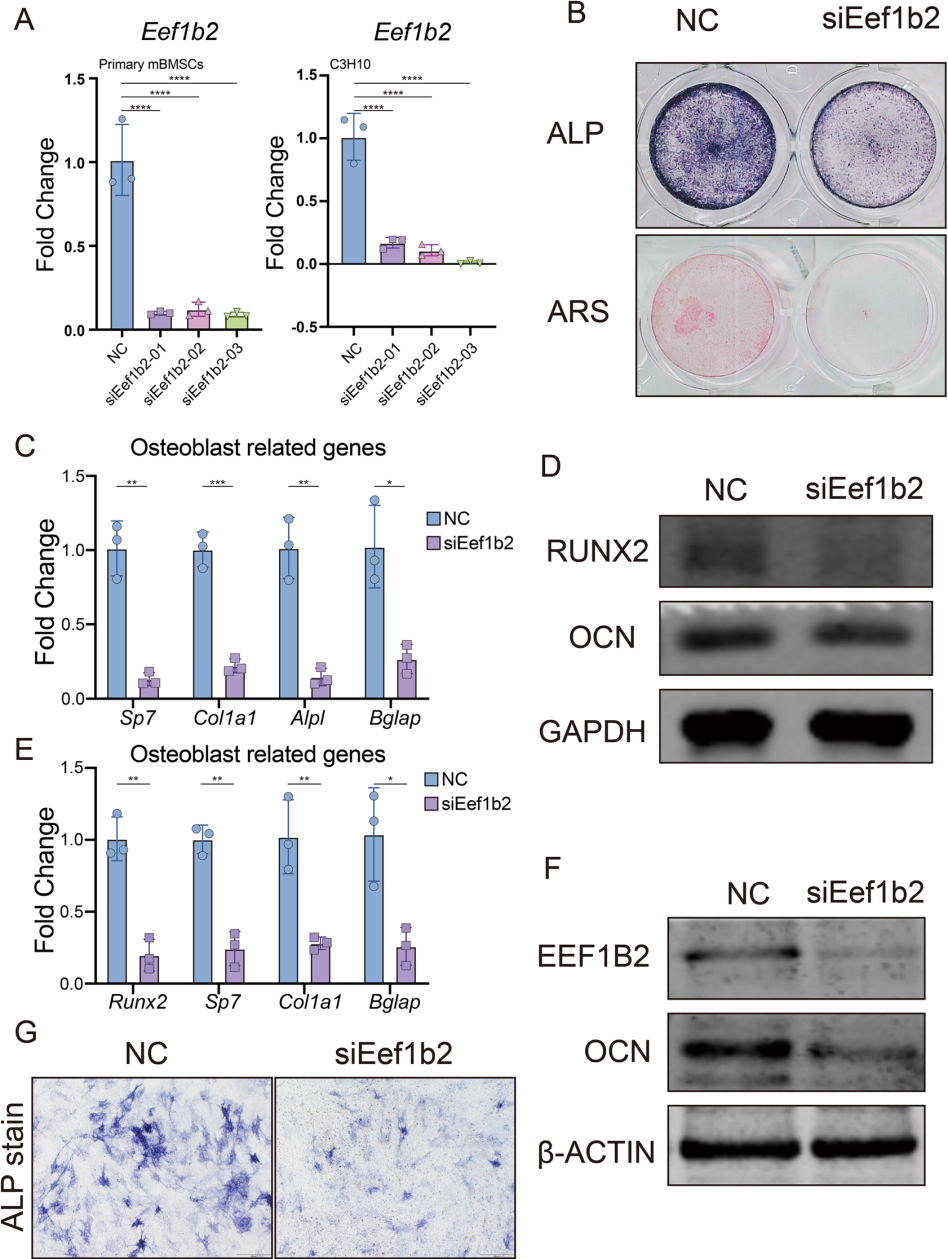
Knockdown EEF1B2 suppresses BMSCs osteogenic differentiation. **(A)** Eef1b2 knockdown efficiency in mouse BMSCs and C3H10T1/2 cells were evaluated by qRT-PCR. **(B)** Osteoblast differentiation and mineralization of mouse BMSCs were accessed by ALP (day 6) and ARS (day 14). **(C)** Relative mRNA expression of osteogenic marker genes *Sp7*, *Col1a1*, *Alpl* and *Bglap* in mouse BMSCs were evaluated by qRT-PCR on day 6 of osteogenic induction. **(D)** Western blot analysis of RUNX2 and OCN protein levels of mouse BMSCs on day 9 under osteogenic induction. **(E)** Relative mRNA expression of osteogenic marker genes *Sp7*, *Runx2*, *Col1a1* and *Bglap* in C3H10T1/2 cells were evaluated by qRT-PCR on day 6 of osteogenic induction. **(F)** Western blot analysis of EEF1B2 and OCN protein levels of C3H10T1/2 cells on day 9 under osteogenic induction. **(G)** Representative images of ALP staining of C3H10T1/2 cell on day 7 under osteogenic induction. Data presented as mean ± SD. *P < 0.05, **P < 0.01, ***P < 0.001, ****P < 0.0001

In order to comprehend the function of EEF1B2 in the process of osteogenic differentiation, we reduce the expression of EEF1B2 in primary mice BMSCs as well as in the murine BMSC cell line C3H10T1/2 cells. The inhibition of *Eef1b2* mRNA by 3 distinct siRNAs was confirmed through qRT-PCR analysis (Fig. 2A). Afterwards, we investigated the potential impact of suppressing EEF1B2 on the process of osteogenic differentiation and mineralization in BMSCs. In the EEF1B2 knockdown (KD) groups, ALP activity significantly decreased after culturing the cells in osteogenic medium (OM) for 7 days (Fig. 2B). Mineralized nodule formation was abolished in EEF1B2 KD groups as shown by Alizarin Red S staining (ARS) (Fig. 2B). In the EEF1B2 KD groups, the expression of *Sp7*, *Col1a1*, *Alpl*, and *Bglap* mRNA was consistently decreased (Fig. 2C). Furthermore, the western blotting analysis demonstrated a reduction in the protein levels of RUNX2 and OCN in the groups with EEF1B2 knockdown (Fig. 2D). Confirming the impairment of osteogenic differentiation, the results were duplicated with C3H10T1/2 cell lines due to the silencing of EEF1B2 (Fig. 2E-G). These data shows that knockdown EEF1B2 expression in BMSCs suppresses osteogenic differentiation.

### Knock down EEF1B2 in BMSCs promote adipogenic differentiation

**Fig. 3:**
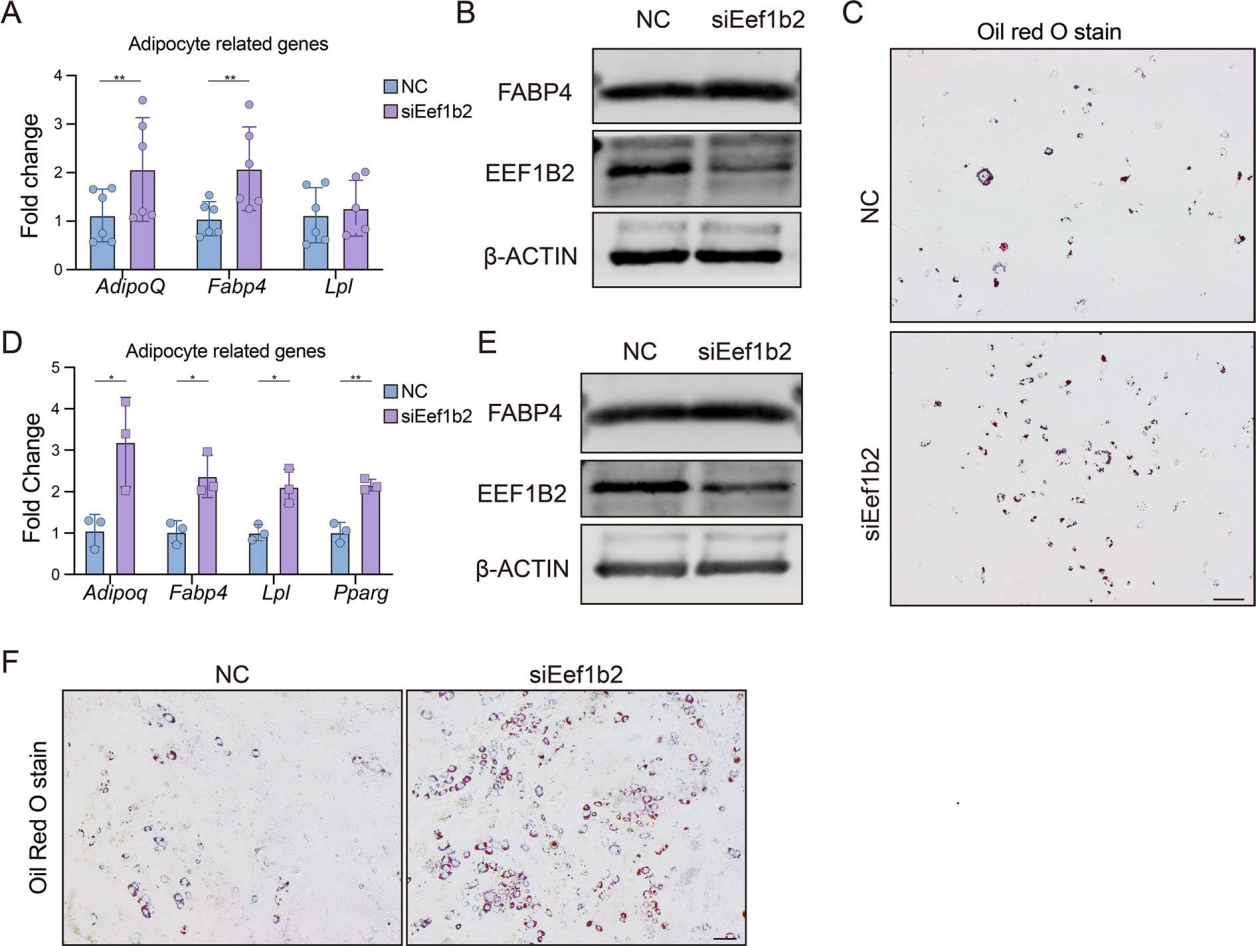
Knockdown EEF1B2 enhances BMSCs adipogenesis. **(A)** Relative mRNA expression of adipogenic marker genes *Adipoq*, *Fabp4* and *Lpl* in mouse BMSCs were evaluated by qRT-PCR on day 4 under adipogenic induction. **(B)** Western blot analysis of FABP4 and EEF1B2 protein levels of mouse BMSCs on day 6 under adipogenic induction. **(C)** Adipogenesis of mouse BMSCs were accessed by Oil Red O staining on day 6 under adipogenic induction. Scale bar, 200 μm. **(D)** Relative mRNA expression of adipogenic marker genes *Adipoq*, *Fabp4*, *Lpl* and *Pparg* in C3H10T1/2 cells were evaluated by qRT-PCR on day 4 under adipogenic induction. **(E)** Western blot analysis of FABP4 and EEF1B2 protein levels of C3H10T1/2 cells on day 6 under adipogenic induction. **(F)** Adipogenesis of C3H10T1/2 cells were accessed by Oil Red O staining on day 6 under adipogenic induction. Scale bar, 200 μm. Data presented as mean ± SD. *P < 0.05, **P < 0.01

Since osteoblast and adipocyte have a shared ancestry, our investigation focuses on determining if reducing EEF1B2 can promote adipogenesis. EEF1B2 silencing led to an augmentation in lipid droplet formation, as evidenced by the intensified staining with Oil Red O (Fig. 3C). Consistently, elevated levels of adipocyte marker gene *Adipoq*, *Lpl*, and *Fabp4* were observed in EEF1B2 KD groups (Fig. 3A). Furthermore, the western blot analysis revealed an elevation in the protein expression of FABP4 in the EEF1B2 KD groups (Fig. 3B). Similarly, knockdown EEF1B2 in C3H10T1/2 cell line under adipogenic medium also showed increased adipogenesis activity (Fig. 3D-F). Our finding indicates that suppressing EEF1B2 expression in BMSCs leads to enhanced adipogenesis.

### EEF1B2 overexpression in C3H10T1/2 cell line promotes osteogenic differentiation and inhibits adipogenic differentiation

**Fig. 4:**
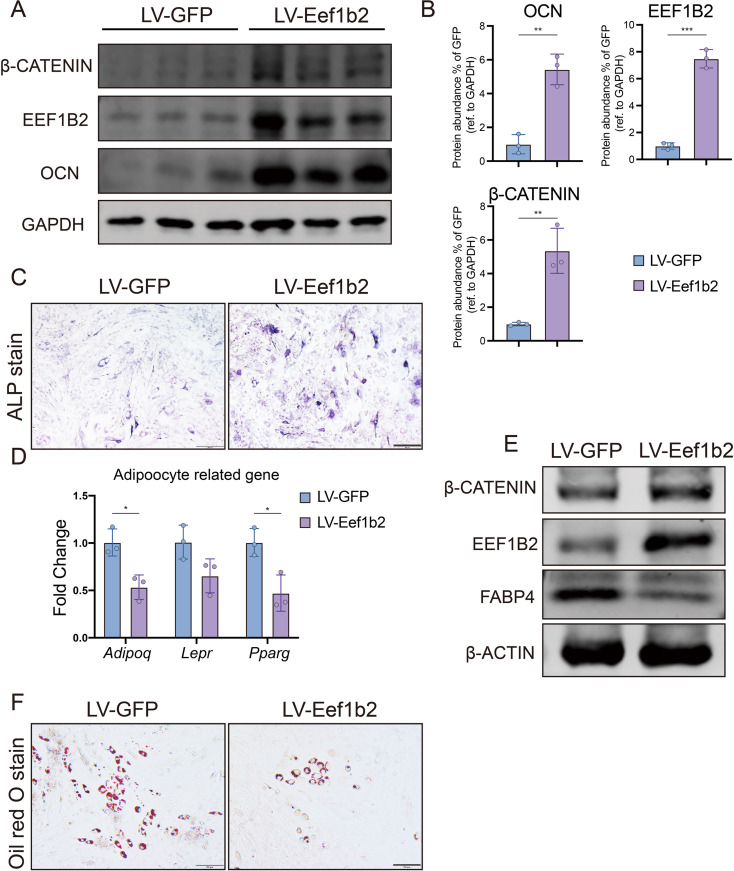
Overexpress EEF1B2 in C3H10T1/2 cells elevates osteogenesis and inhibits adipogenesis. **A-B** Representative images (**A**) and quantitative data (**B**) of western blot analysis of β-catenin, EEF1B2, OCN protein levels of C3H10T1/2 cells on day 9 under osteogenic induction. **C.** Representative images of ALP staining of C3H10T1/2 cell on day 7 under osteogenic induction. Scale bar, 200 μm. **D.** Relative mRNA expression of adipogenic marker genes *Adipoq*, *Lepr* and *Pparg* in C3H10T1/2 cells were evaluated by qRT-PCR on day 4 under adipogenic induction. **E.** Western blot analysis of β-catenin, EEF1B2 and FABP4 protein levels of C3H10T1/2 cells on day 6 under adipogenic induction. **F.** Representative images of Oil Red O staining of C3H10T1/2 cell on day 6 under adipogenic induction. Scale bar, 100 μm. Data presented as mean ± SD. *P < 0.05, **P < 0.01, ***P < 0.001

In order to validate the impact of EEF1B2 on the cellular destiny of BMSCs, lentivirus was utilized to overexpress EEF1B2 in the C3H10T1/2 cell line. The Western blot analysis revealed a significant increase in the expression of EEF1B2 (Fig. 4A, B). By contrast, ALP activity was enhanced when EEF1B2 was overexpressed (Fig. 4C).

To explore the impact of EEF1B2 overexpression on the adipogenic differentiation of C3H10T1/2, we investigated its potential contrary effect on osteogenesis and adipogenesis caused by EEF1B2 knockdown. EEF1B2 overexpression groups exhibited a decrease in lipid droplet formation, as evidenced by the reduced staining of Oil Red O (Fig. 4F). Furthermore, the mRNA expression of the adipogenic marker genes were reduced when EEF1B2 was overexpressed (Fig. 4D), and down-regulated of FABP4 in LV-Eef1b2 group was shown by western blot analysis (Fig. 4E). In summary, our results indicate that increased expression of EEF1B2 promotes osteogenic differentiation while inhibiting adipogenic differentiation in the C3H10T1/2 cell line.

### EEF1B2 overexpression mitigates bone loss and diminishes marrow adiposity in mice afflicted with osteoporosis

**Fig. 5:**
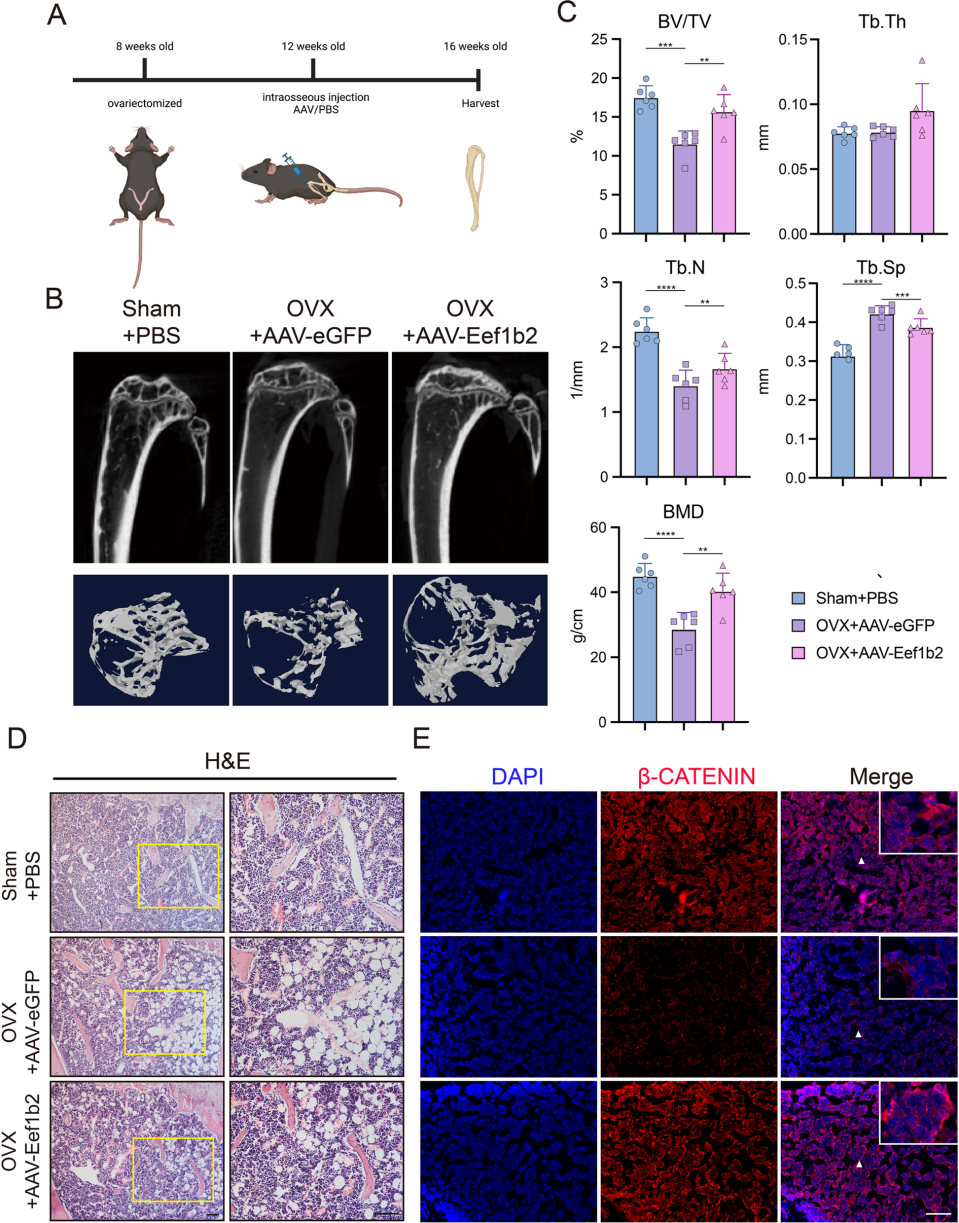
Overexpress EEF1B2 mitigates bone loss and diminishes marrow adiposity in mice afflicted with osteoporosis. **A.** A schematic map of experiment process. **B-C.** Representative images (**B**) and quantitative data (**C**) of micro-CT analysis of tibias from different groups of mice. **D.** Representative images of H&E staining of proximal tibia from different groups of mice. Scale bar, 200 μm. **E.** β-catenin expression in tibias was evaluated by immunofluorescence. Scale bar, 200 μm. Data presented as mean ± SD. *P < 0.05, **P < 0.01, ***P < 0.001, ****P < 0.0001

In order to elucidate the function of EEF1B2 in the determination of BMSCs cell destiny in vivo, a model of osteoporosis was generated through the removal of ovaries in mice (OVX mice). AAV was administered into the marrow cavities of both tibias in OVX mice (Fig. 5A). After 4 weeks of injection, the microtomography analysis (micro-CT) revealed a rise in BV/TV, BMD, Tb.N, and a decrease in Tb.Sp in the OVX + AAV-Eef1b2 group when compared to the OVX + AAV-eGFP group (Fig. 5B, C). This suggests that the overexpression of EEF1B2 in the bone marrow cavities of OVX mice can alleviate bone loss. Furthermore, the H&E staining results indicate that OVX mice treated with AAV-Eef1b2 exhibited a decrease in adipocytes in comparison to the OVX + AAV-eGFP groups (Fig. 5C). These findings highlight the importance of EEF1B2 overexpression in bone marrow in mitigating bone loss and decreasing marrow fat in mice that have undergone ovariectomy.

### EEF1B2 controls the transition from adipogenic to osteogenic differentiation of BMSCs through the Wnt/β-catenin signaling pathway

**Fig. 6:**
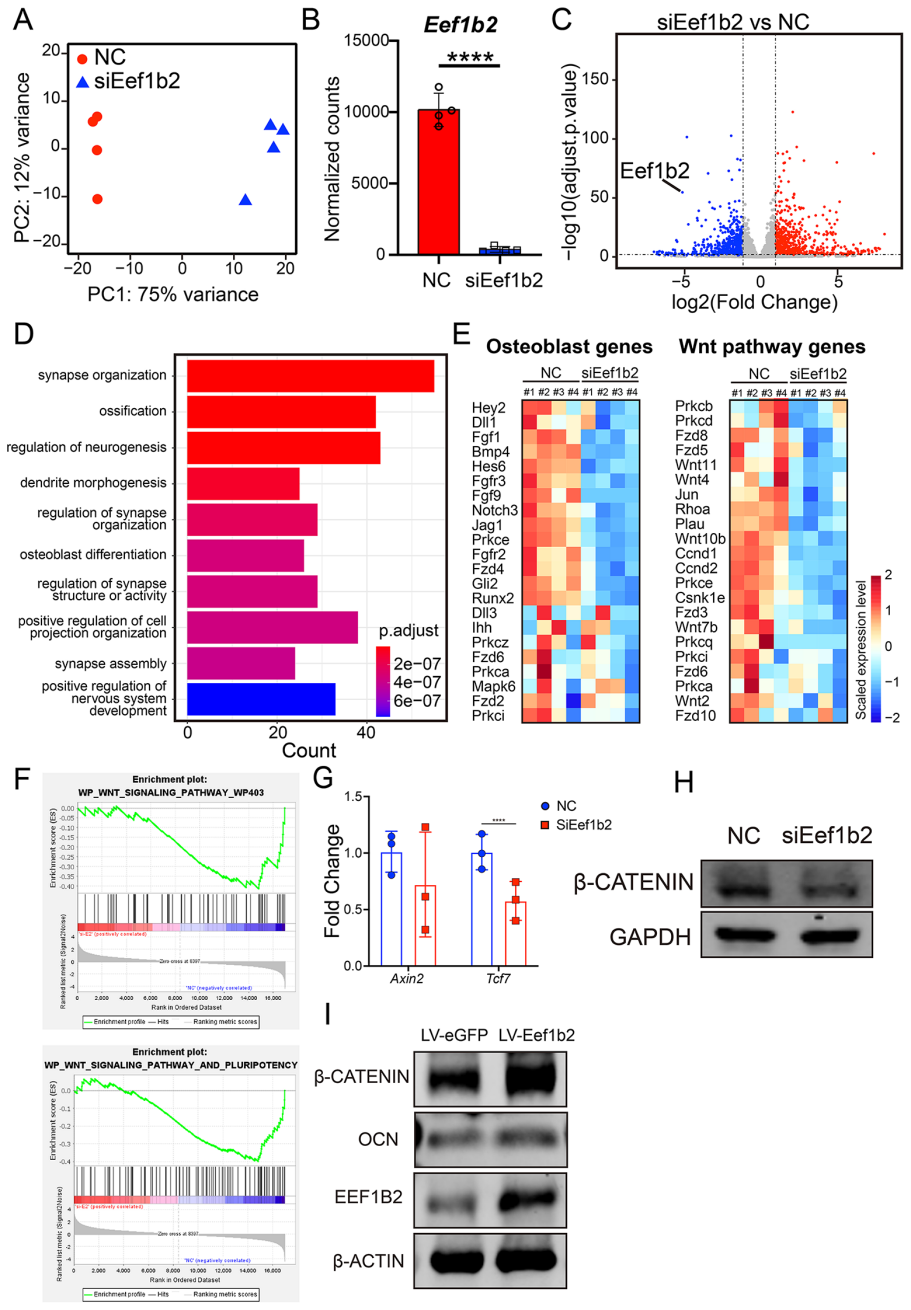
EEF1B2 regulates BMSCs osteogenesis and adipogenesis by modulating Wnt/β-catenin signaling activity. **(A)** Principal component analysis (PCA) of the transcriptomes of NC and siEef1b2 groups (*n* = 4 per group). **(B)** Normalized counts of Eef1b2 in siEef1b2 groups compared to NC groups. ****p value < 0.0001. **(C)** Volcano plot exhibit the DEGs of the siEef1b2 group compared to the NC group (*n* = 4 per group). Blue dots show genes more highly expressed in the NC group. Red dots show genes more highly expressed in the siEef1b2 group. **(D)** GO enrichment analysis of DEGs upregulated in the NC group. **(E)** Scaled expression levels of selected osteoblast differentiation or Wnt pathway related genes was showed by heatmap. **(F)** Gene set enrichment analysis (GSEA) shows the enrichment score of Wnt signaling pathway by comparing with the siEef1b2 group to the NC group. (Wnt signaling pathway, NES=-1.546, Nominal p-value = 0.025; Wnt signaling pathway and pluripotency, NES=-1.600, Nominal p-value = 0.004. **(G)** Relative mRNA expression of β-catenin readout genes *Tcf7* and *Axin2*. **(H)** Western blot analysis of β-catenin protein level of mouse BMSCs on day 9 under osteogenic induction. **(I)** Western blot analysis of β-catenin, OCN and EEF1B2 protein level of C3H10T1/2 cells on day 9 under osteogenic induction. Data presented as mean ± SD. *P < 0.05

To investigate the underlying mechanism by which EEF1B2 controls the cell destiny of BMSCs, bulk RNA sequencing (RNA-seq) analysis was performed on BMSCs treated with or without siRNA targeted to *Eef1b2* during osteogenic induction. Four biological repeated experiments were carried out in each group. Principal component analysis (PCA) exhibited well distinguished gene expression features between negative control (NC) and siEef1b2 group(Fig. 6A). Normalized counts of the gene *Eef1b2* verified the significant knock down efficiency of the siRNA treated group in the RNA-seq data (Fig. 6B). Differentially expressed genes (DEGs) were defined as genes with Benjamini-Hochberg’s false discovery rate (FDR) < 0.05 and fold change > 2 between two groups and visualized via volcano plot (Fig. 6C). Gene ontology (GO) enrichment analysis using downregulated DEGs in siEef1b2 group as input showed the enriched biological processed including ossification and osteoblast differentiation (Fig. 6D). The gene expression values of the most enriched Wnt pathway correlated gene set and the osteoblast gene set from NC BMSC RNA-seq data were extracted and displayed in the heatmaps (Fig. 6E). Gene set enrichment analysis (GSEA) revealed the significantly enriched gene set to be Wnt signaling pathway and pluripotency (*p* < 0.01) and Wnt signaling pathway (*p* < 0.05) in NC group compared to siEef1b2 group (Fig. 6F). Consistently, the results of qRT-PCR demonstrated a decrease in the expression of the Tcf7 gene, which is a readout gene for β-catenin. The Western blot analysis demonstrated that the expression of β-catenin was reduced in groups where EEF1B2 was knocked down (Fig. 6H) and increased in the group with overexpression of EEF1B2 (Figs. 4A and E and 6I). Furthermore, the tibias of OVX + AAV-eGFP mice exhibited a decrease in β-catenin expression when compared to the Sham + PBS groups. The overexpression of EEF1B2 led to a significant increase in the β-catenin level within the tibia cavities (Fig. 5D). These data support that EEF1B2 regulates BMSCs osteogenesis and adipogenesis by modulating Wnt/β-catenin signaling activity.

**Fig. 7:**
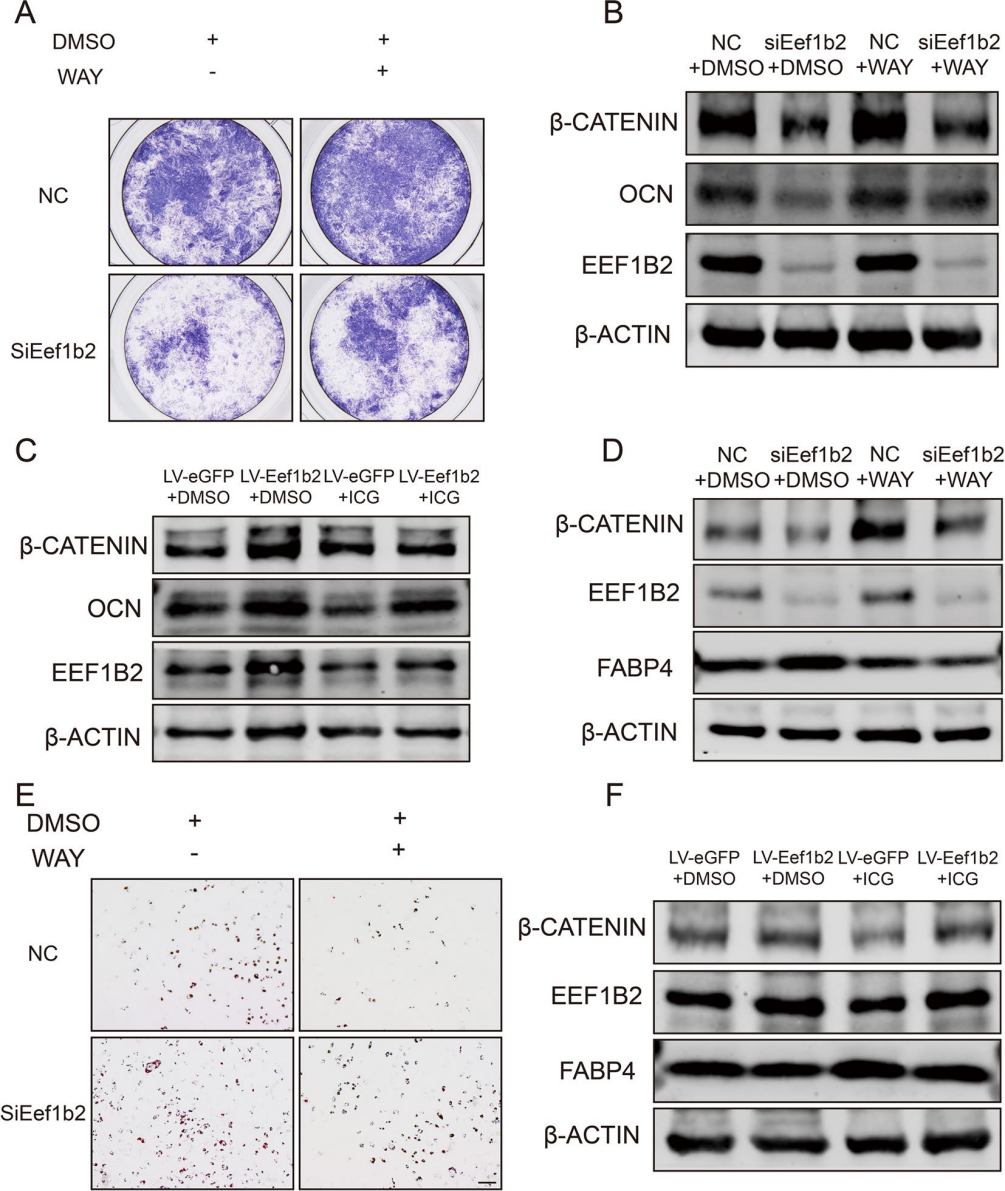
Activate or inhibit β-catenin reverses the effect of EEF1B2 silencing or overexpression in osteogenesis and adipogenesis in BMSCs. (A) Representative image of ALP staining of mouse BMSCs on day 6 under osteogenic differentiation. (B) Western blot analysis of β-catenin, OCN and EEF1B2 protein level of mouse BMSCs on day 9 under osteogenic induction. (C) Western blot analysis of β-catenin, OCN and EEF1B2 protein level of C3H10T1/2 cells on day 9 under osteogenic induction. (D) Western blot analysis of β-catenin, FABP4 and EEF1B2 protein level of mouse BMSCs on day 6 under adipogenic induction. (E) Adipogenesis of mouse BMSCs were accessed by Oil Red O staining on day 6 under adipogenic induction. Scale bar, 200 μm. (F) Western blot analysis of β-catenin, FABP4 and EEF1B2 protein level of C3H10T1/2 cells on day 6 under adipogenic induction

To further investigate the role of β-catenin in mediating EEF1B2-regulated BMSCs differentiation, we treated EEF1B2 KD BMSCs and the control group with WAY-262,611, a β-catenin agonist. β-catenin activation could partially rescue the inhibition of osteogenesis and promotion of adipogenesis by EEF1B2 silencing (Fig. 7A, B, D, E). In contrast, β-catenin inhibition eliminates the positive role of overexpression of EEF1B2 in osteogenic and adipogenic differentiation of C3H10T1/2 cells (Fig. 7C, F). Taken together, these data show that EEF1B2 regulates β-catenin activity to restore the equilibrium between osteogenesis and adipogenesis, suggesting the involvement of Wnt/β-catenin signaling in the osteogenic and adipogenic differentiation of BMSCs during the progression of osteoporosis.

## Discussion

Bone marrow-derived mesenchymal stem cells have the ability to transform into various types of cells, such as bone-forming cells and fat cells. Therefore, the differentiation direction of BMSCs is deemed crucial for the study and treatment of osteoporosis. Adipocytes accumulation and osteoblasts reduction are of high relevance to osteoporosis [43]. Currently, a significant body of research has examined the regulatory mechanisms governing the unidirectional differentiation of BMSCs towards osteogenic or adipogenic lineages. Some studies have also explored the involvement of BMSCs in maintaining the balance between bone and fat. Nevertheless, there is a lack of publicly available reports on the effects and underlying mechanisms of EEF1B2 on the directional differentiation of BMSCs. In our present investigation, we have shown that the involvement of EEF1B2 in the transition from osteogenesis to adipogenesis in BMSCs is evident as the inhibition of EEF1B2 suppresses BMSCs’ ability to form bone but promotes the formation of fat cells. In terms of mechanism, we discovered that EEF1B2 facilitated β-catenin activity, thereby regulating the differentiation of BMSCs. These results demonstrated the integral role of EEF1B2 in BMSCs differentiation.

The process of protein synthesis in eukaryotes is dependent on a tightly controlled mechanism that involves initiation, elongation, and termination [44]. To ensure the precise production of the protein at the correct time and location, every stage of this procedure is meticulously regulated [45]. The regulation of gene expression in transcriptional control is widely acknowledged, although there remain numerous unresolved aspects regarding the regulation at the translation level [46]. Lately, there has been an increasing amount of proof regarding further regulation during the elongation stage. There are three types of supramolecular complexes involved in elongation in eukaryotes: the ribosome, the complex of elongation factors, and the multienzyme aminoacyl-tRNA synthetase complex [47]. The aminoacyl-tRNA delivery step is catalyzed by the eEF1 complex. The eEF1 family includes eEF1A and eEF1B [48]. The canonical role of eEF1B is to ensure guanine nucleotide exchange on eEF1A during the elongation process [49]. In addition to its role in translation elongation, eEF1B has also been associated with overexpression in lung cancer, indicating its involvement in tumorigenesis [19]. Furthermore, according to the report, eEF1B is extensively expressed in various cell lines and tissues, indicating its crucial involvement in gene expression [18]. According to our data, EEF1B2 is detected in mouse primary BMSCs and the C3H10T1/2 cell line. It was found to be increased during osteogenic differentiation but decreased during adipogenic differentiation, suggesting a possible involvement in the regulation of cell fate. Further experiments involving knocking down and overexpressing EEF1B2 provided additional clarification on its role in regulating the differentiation of BMSCs, specifically in the opposite directions of osteogenesis and adipogenesis.

Cell differentiation, proliferation, and migration are crucial functions regulated by the Wnt/β-catenin signaling pathway [50]. The regulation of osteogenesis and adipogenesis by Wnt/β-catenin is widely recognized as crucial for maintaining bone equilibrium. The activation of the Canonical Wnt signaling pathway relies on the activity of the transcription factor β-catenin [51]. When β-catenin accumulates in the nucleus, it forms complexes with LEF/TCF transcription factors to initiate the transcription of downstream target genes, such as Sp7 and Runx2. Conversely, suppression of β-catenin leads to the upregulation of the critical adipogenic transcription factors C/EBPα and PPARγ to promote adipogenesis [27, 52]. Previous research has indicated that, in addition to the Wnt family members, various elements such as ANKRD1, SFRPs, and PTTG1 have the ability to regulate the activation of β-catenin [53–55]. During the osteogenic differentiation condition, we conducted bulk RNA sequencing on BMSCs with EEF1B2 knockdown and a negative control group in the present investigation. The GSEA examination revealed a modification in the Wnt/β-catenin signaling pathway between the aforementioned groups. Our study explored the influence of decreased EEF1B2 expression on the Wnt/β-catenin signaling pathway during the osteogenic differentiation of BMSCs, considering the associations uncovered through RNA sequencing analysis. The downregulation of EEF1B2 inhibited osteogenic differentiation of BMSCs through modulation of the Wnt/β-catenin signaling pathway. As the Wnt/β-catenin signaling pathway acts as a switch in BMSCs cell fate choice, we wondered whether adipocytes accumulation in EEF1B2 knock down BMSCs is caused by β-catenin suppression. The results showed a reduced β-catenin activity in BMSCs undergoing adipogenic differentiation upon EEF1B2 knockdown. Notably, the expression of EEF1B2 remained unaltered following treatment with either a Wnt agonist or antagonist. These findings suggest that EEF1B2 is one of the regulators of the Wnt/β-catenin signaling pathway during BMSCs differentiation. It is important to note that this study solely demonstrates the ability of EEF1B2 to regulate β-catenin activity during BMSCs osteogenic and adipogenic differentiation, and therefore it remains to be determined how EEF1B2 modulates Wnt/β-catenin signaling pathway.

Discovering an alternative method to promote osteogenic differentiation and suppress adipogenic differentiation of BMSCs proves to be a successful approach in the treatment of osteoporosis [56, 57]. This study has provided confirmation that the overexpression of EEF1B2 has the potential to enhance bone formation and decrease the accumulation of fat in the bone marrow of OVX mice, offering valuable insights for the treatment of osteoporosis.

To summarize, this research discovered that EEF1B2 functions as a toggle to regulate the fate determination of BMSCs and maintain a balance between bone and fat by controlling the Wnt/β-catenin signaling pathway. It shows immense potential as a possible treatment approach for bone metabolic disorders.

**Table 1:** Primer sequence

S. No.	Gene name (Mus musculus)	Direction	Sequence
1	Eef1b2	Forward primer	5’-TGACCTGTGTCATGCCCTAC-3’
		Reverse primer	5’-GCCATACTTGCCCAAAGATTTCT-3’
2	Runx2	Forward primer	5’-AACCCACGGCCCTCCCTGAACTCT-3’
		Reverse primer	5’-ACTGGCGGGGTGTAGGTAAAGGTG-3’
3	Sp7	Forward primer	5’-CCCACTGGCTCCTCGGTTCTCTCC-3’
		Reverse primer	5’-GCTBGAAAGGTCAGCGTATGGCTTC-3’
4	Col1a1	Forward primer	5’-CACCCTCAAGAGCCTGAGTC-3’
		Reverse primer	5’-GTTCGGGCTGATGTACCAGT-3’
5	Bglap	Forward primer	5’-ACCCTGGCTGCGCTCTGTCTCT-3’
		Reverse primer	5’-GATGCGTTTGTAGGCGGTCTTCA-3’
6	Alpl	Forward primer	5’-CTTGACTGTGGTTACTGCTGAT-3’
		Reverse primer	5’-GGAATGTAGTTCTGCTCATGGA-3’
7	Tcf7	Forward primer	5’-TCGAGAAGAGCAGGCCAAGT-3’
		Reverse primer	5’-AGAGCACTGTCATCGGAAGGAA-3’
8	Axin2	Forward primer	5’-CCATTGGAGTCTGCCTGTG-3’
		Reverse primer	5’-GGACACTTGCCAGTTTCTTTG-3’
9	Pparg	Forward primer	5’-AAGAAGCGGTGAACCACTGA-3’
		Reverse primer	5’-TGCGAGTGGTCTTCCATCAC-3’
10	Adipoq	Forward primer	5’-CCAATGTACCCATTCGCTTTAC-3’
		Reverse primer	5’-GAAGTAGTAGAGTCCCGGAATG-3’
11	Cebpa	Forward primer	5’-GCGGGAACGCAACAACATC-3’
		Reverse primer	5’-GTCACTGGTCAACTCCAGCAC-3’
12	Fabp4	Forward primer	5’-TCATAACCCTAGATGGCGGGG-3’
		Reverse primer	5’-GCCTTTCATAACACATTCCACCA-3’
13	Lpl	Forward primer	5’-TTGGAGAAGCTATCCGCGTG-3’
		Reverse primer	5’-CGTGGGAGCACTTCACTAGC-3’
14	Lepr	Forward primer	5’-GAAAAATGGATGGGGACGTTAC-3’
		Reverse primer	5’-CAGTGAGTCATTTTTCGTCAGG-3’
15	β-Actin	Forward primer	5’-TCCGGCACTACCGAGTTATC-3’
		Reverse primer	5’-GATCCGGTGTAGCAGATCGC-3’
16	Gapdh	Forward primer	5’-ATCAAGAAGGTGGTGAAGCA-3’
		Reverse primer	5’-AGACAACCTGGTCCTCAGTGT-3’

**Table 2:** Antibodies

No.	Antibodies	Sources	Cat. No.	Dilution	Applications
1	Anti-EEF1B2	Proteintech	10483-1-AP	1:1000	WB
2	Anti-β-ACTIN	Proteintech	66009-1-Ig	1:10000	WB
3	Anti-β-CATENIN	Proteintech	51067-2-AP	1:5000	WB
4	Anti-FABP4	Proteintech	12802-1-AP	1:5000	WB
5	Anti-GAPDH	Santa Cruz	sc-365,062	1:3000	WB
6	Anti-RUNX2	Beyotime	AF2593	1:500	WB
7	Anti-Osteocalcin	Abcam	ab93876	1:500	WB
8	Anti-β-CATENIN	Proteintech	51067-2-AP	1:200	IF
9	Goat anti-Mouse IgG H&L (IRDye® 800CW)	Abcam	ab216772	1:5000	WB
10	Goat anti-Rabbit IgG H&L (IRDye® 800CW)	Abcam	ab216773	1:5000	WB
11	Goat Anti-Rabbit IgG H&L (Alexa Fluor® 555)	Abcam	ab150078	1:500	IF

## Data Availability

The bulk RNA-Seq data in this study have been deposited Genome Sequence Archive (GSA) under accession number PRJCA026878.

## Abbreviations

BMSCs: Bone marrow-derived mesenchymal stem cells
EEF1B2: Eukaryotic translation elongation factor 1 beta 2
OCN: Osteocalcin
IBMX: 3-Isobutyl-1-methylxanthine
BSA: Bovine serum albumin
siRNA: Small interfering RNA
OM: Osteogenic medium
ALP: Alkaline phosphatase
ARS: Alizarin Red S
KD: Knock down
OVX: Ovariectomy
LV: Lentivirus
AAV: Adeno-associated virus
DAPI: 4’,6-diamidino-2-phenylindole
PCA: Principal component analysis
DEGs: Differentially expressed genes
GO: Gene ontology
GSEA: Gene set enrichment analysis
BV/TV: Trabecular bone volume to total volume
BMD: Bone mineral density
Tb.N: Trabecular number
Tb.Sp: Trabecular separation
